# White blood cell count profiles in multiple sclerosis during attacks before the initiation of acute and chronic treatments

**DOI:** 10.1038/s41598-021-01942-8

**Published:** 2021-11-16

**Authors:** Tetsuya Akaishi, Tatsuro Misu, Kazuo Fujihara, Naoki Nakaya, Tomohiro Nakamura, Mana Kogure, Rieko Hatanaka, Fumi Itabashi, Ikumi Kanno, Toshiyuki Takahashi, Hiroshi Kuroda, Juichi Fujimori, Yoshiki Takai, Shuhei Nishiyama, Kimihiko Kaneko, Tadashi Ishii, Masashi Aoki, Ichiro Nakashima, Atsushi Hozawa

**Affiliations:** 1 Department of Neurology, Tohoku University Graduate School of Medicine, Seiryo-machi 1-1, Aoba-ku, Sendai, Miyagi 980-8574 Japan; 2 Department of Education and Support for Regional Medicine, Tohoku University Hospital, Sendai, Japan; 3 Department of Multiple Sclerosis Therapeutics, Fukushima Medical University, Fukushima, Japan; 4 Tohoku Medical Megabank Organization, Tohoku University, Sendai, Japan; 5 Department of Neurology, National Hospital Organization Yonezawa National Hospital, Yonezawa, Japan; 6 Department of Neurology, Tohoku Medical and Pharmaceutical University, Sendai, Japan

**Keywords:** Neurology, Neurological disorders

## Abstract

Multiple sclerosis (MS) is a major demyelinating disease of the central nervous system; however, its exact mechanism is unknown. This study aimed to elucidate the profile of white blood cells (WBCs) in the acute phase of an MS attack. Sixty-four patients with MS at the time of diagnosis and 2492 age- and sex-adjusted healthy controls (HCs) were enrolled. Data regarding the blood cell counts were compared between the groups. The total WBC (*p* < 0.0001), monocyte (*p* < 0.0001), basophil (*p* = 0.0027), and neutrophil (*p* < 0.0001) counts were higher in the MS group than in the HC group, whereas the lymphocyte and eosinophil counts did not differ. Adjustments for the smoking status and body mass index yielded the same results. The total and differential WBC counts of the patients with MS did not correlate with the counts of T2 hyperintense brain lesions or the levels of neurological disturbance. In summary, patients with MS showed elevated counts of total WBCs, monocytes, basophils, and neutrophils at the time of diagnosis. However, the clinical relevance of these biomarkers in the context of the development and progression of MS remains unclear.

## Introduction

Multiple sclerosis (MS) is an inflammatory neurological disease characterized by demyelinating lesions of the central nervous system (CNS) and typically presenting with recurrent neurological episodes, with or without the indolent progression of neurological disturbances between relapses^1–3^. Although the disease is believed to be an autoimmune-related neurological disorder, the exact mechanisms, which are thought to be multifactorial, have not yet been fully elucidated. Furthermore, the white blood cell (WBC) profiles in MS, as opposed to those in healthy controls (HCs), have not yet been largely examined. The WBC differential count is a popular biomarker for evaluating the presence of systemic inflammations^4^. Several previous studies have reported that patients with MS may show slightly elevated levels of neutrophil-to-lymphocyte ratios (NLR) in the peripheral blood compared to HCs, which may reflect the disease activity and prognosis^4–7^. This fact suggested that the innate immune system, rather than the adaptive immune system, might be activated in patients with MS^8,9^. The WBC differential count is an inexpensive and easily accessible biomarker, and hence can help in the diagnosis and treatment of the patients with MS. This study aimed to confirm the activation of the innate immune system in MS by enrolling a large number of HCs, and further attempted to determine the influence of differential WBC counts on the clinical manifestation of the disease.

## Methods

### Study design

This study was a single-center retrospective case–control study. All consecutive patients who were diagnosed with MS as per the previous or latest diagnostic criteria^3,10^, and had hemogram data at the time of diagnosis before starting acute or chronic treatment (i.e., disease-modifying drugs) were enrolled. All enrolled patients were reconfirmed to meet the latest version of the McDonald diagnostic criteria as revised in 2017^3^. All enrolled patients with MS were treated at Tohoku University Hospital. Patients whose conditions remained as clinically isolated syndromes or radiologically isolated syndromes and did not progress to MS by March 2021 were not recruited. A sufficiently large number of HCs in their 20s, 30s, and 40s, with no significant medical history, were also enrolled to compare the hemogram data. The profile of HCs has previously been reported^11,12^. Patients with MS whose hemogram data at the diagnosis were achieved before April 2013, when the automated hematology analyzer was changed to XN-Series at the hospital, were not enrolled for this study to avoid biases based on the measurement with different analyzers.

### Evaluated Variables

The background and hemogram data of the evaluated participants were collected, and all the blood samples were collected and evaluated at a single university in Japan. Blood samples from patients with MS were collected at the time of diagnosis before they underwent acute treatment with intravenous methylprednisolone pulse therapy, or relapse prevention treatment. Age and sex data were evaluated to attain background information. Data regarding the current smoking status, previous smoking status, and body mass index (BMI) were also collected, as the current smoking status and obesity are known to affect the total and differential WBC counts.

Hemograms of all the participant samples were generated automatically using XN-Series systems (Sysmex, Kobe, Japan). The total WBC, red blood cell (RBC), and platelet counts in the peripheral blood were collected for both the groups (HC and MS). Furthermore, the differential counts of monocytes, lymphocytes, eosinophils, basophils, and neutrophils were determined, and the NLR, platelet-to-lymphocyte ratio (PLR), and monocyte-to-lymphocyte ratio (MLR) were derived from each component.

In addition to the blood cell count data, other blood test data, cerebrospinal fluid (CSF) analysis data, and clinical variables regarding disease activity and clinical severity were collected from the MS group. Additional blood test data included the serum C-reactive protein (CRP) level, anti-nuclear antibody (ANA; ≥ 1:160) positivity, presence of hepatitis B surface antigen (HBsAg), presence of antibodies against hepatitis C virus (HCV), and the results of *Treponema pallidum* hemagglutination (TPHA) testing for syphilis screening. The CSF analysis data included the total cell count (CSF-CC), presence of CSF-restricted oligoclonal bands (OCB), immunoglobulin-G index (IgG-index), CSF/serum ratios of albumin (QAlb), and CSF/serum ratios of IgG (QIgG). The outcome variables included the count of T2 hyperintense demyelinating lesions on brain MRI, regardless of the contrast enhancement at the time of hemogram, and the scores of the Kurtzke’s Expanded Disability Status Scale (EDSS) during the remission phase 1 year after the hemogram.

### Statistical Analysis

Distributions of quantitative variables were described as the median and interquartile range (IQR, 25–75 percentiles). The Mann–Whitney U test was used to compare the quantitative variables between the two independent groups. As the total and differential WBC counts are known to be largely affected by the current smoking status and BMI, the analysis of covariance (ANCOVA) for each of the WBC differentials were performed by using the BMI and average number of cigarettes smoked per day as covariates. Correlation coefficients between the two variables were evaluated by calculating the Pearson’s correlation coefficient (R) or Spearman’s correlation coefficient (rho) according to the distribution patterns of the evaluated variables, which was followed by a test of no correlation. Hierarchical clustering was performed to categorize the five WBC subtypes and generate dendrograms. Statistical significance was set at *p* < 0.005 for simultaneous multiple comparisons with the Bonferroni adjustment. Statistical analyses were performed using the R Statistical Software (version 4.0.5; R Foundation, Vienna, Austria). The Cocor package was used to compare the R values of the two independent groups^13^.

### Ethics

This study was approved by the Institutional Review Board of the Tohoku University School of Medicine (IRB approval number: 2020-4-072). All processes in this study were performed in accordance with the current version of the Declaration of Helsinki, as revised in 2013. Written informed consent was obtained from all enrolled individuals.

## Results

### Participants

A total of 64 patients with MS (13 men and 51 women) and 2492 HCs without any neurological disease (638 men and 1854 women) were enrolled. Among the 64 patients with MS, 62 had relapsing–remitting MS and two had primary progressive MS. Thirty-two of the patients were with acute myelitis with objective clinical evidence, while the other 32 patients were in the absence of acute myelitis at the time of diagnosis. The demographic features and blood test data of the MS and HC groups are summarized in Table 1.

**Table 1:** Demographic and laboratory features of the patients with multiple sclerosis and healthy controls.

	Patients with MS	HC	*p* value
n	64	2492	–
Male, n (%)	13 (20.3%)	638 (25.6%)	0.3376
Age*	34 (28–43) years	35 (32–38) years	0.4335
Current smoker, n (%)	11/61 (18.0%)	562/2485 (22.6%)	0.3971
Ex-smoker, n (%)	4/61 (6.6%)	554/2485 (22.3%)	0.0016
Body-mass index	21.8 (19.7–24.3)	21.4 (19.5–24.0)	0.4435
Total WBC count [/μL]*	6300 (5500–7400)	5400 (4500–6500)	< 0.0001
RBC count [× 10^6^/μL]*	4.65 (4.43–4.86)	4.53 (4.29–4.83)	0.0260
Platelet count [× 10^3^/μL]*	272 (238–304)	251 (217–289)	0.0155
Monocyte [/μL]*	315 (238–380)	258 (202–323)	< 0.0001
Lymphocyte [/μL]*	1810 (1480–2290)	1770 (1480–2130)	0.5025
Eosinophil [/μL]*	135 (80–240)	120 (70–200)	0.3523
Basophil [/μL]*	40 (20–50)	30 (20–40)	0.0027
Neutrophil [/μL]*	3740 (3000–4750)	3100 (2440–3980)	< 0.0001
PLR*	143 (113–186)	140 (114–174)	0.3142
MLR*	0.166 (0.142–0.201)	0.142 (0.113–0.180)	0.0003
NLR*	2.10 (1.49–3.24)	1.75 (1.37–2.23)	0.0029

The *p* values were calculated using the chi-square test for sex ratio and current smoker ratio, Fisher’s exact test for ex-smoker ratio, and the Mann–Whitney U test for other variables. The total WBC, monocyte, basophil, and neutrophil counts were significantly higher in the MS group than in the HC group.

HC, healthy control; MLR, monocyte-to-lymphocyte ratio; MS, multiple sclerosis; NLR, neutrophil-to-lymphocyte ratio; PLR, platelet-to-lymphocyte ratio; RBC, red blood cell; WBC, white blood cell count.

*Median and interquartile range (25–75 percentiles).

Among the 64 patients with MS, 54 were tested for the presence of HBsAg, antibodies against HCV, and TPHA testing, and none of them tested positive for any of these antibodies. IgM antibodies against other infections, such as measles, rubella, mumps, varicella-zoster virus (VZV), herpes simplex virus (HSV), cytomegalovirus, and human immunodeficiency virus (HIV), were absent in all of the evaluated patients with MS. Fifty-eight of the 64 patients with MS were evaluated for ANA positivity; of them, 57 (98.3%) tested negative (1:40), and one patient presented a value of 1:160. Serum CRP level was evaluated in all 64 patients, of whom 62 (96.9%) revealed normal levels (< 0.30 mg/dL) and two patients revealed a slightly elevated level (0.38 mg/dL and 0.30 mg/dL). Fifty-seven of the 64 patients underwent CSF analysis at the time of hemogram, and CSF-restricted OCBs were positive in 47 (82.5%) patients. The median (IQR) for CSF-CC was 2 (1–5) [/μL], for IgG-index was 0.68 (0.60 – 0.89), for QAlb was 0.0048 (0.0034–0.0059), and for QIgG was 0.0035 (0.0025–0.0048).

### Blood cell counts in the HC and MS groups

**Figure 1:**
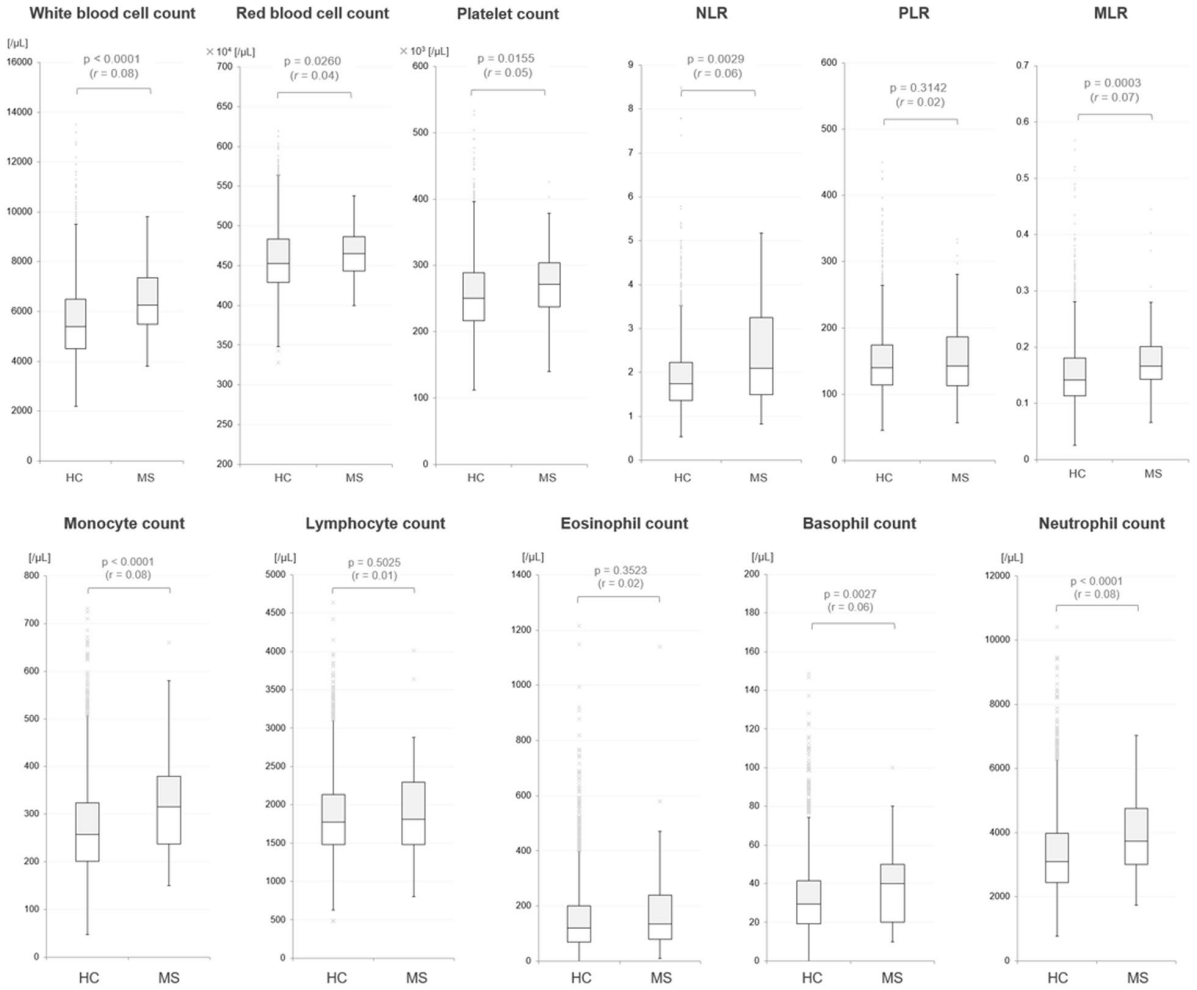
Boxplots of blood cell subtypes in the HC and MS Groups. Boxplots for three blood cell types, five differential white blood cell subtypes, and the three derived ratios (NLR, PLR, MLR) calculated from the differential white blood cell counts are shown. The p-values are the results of the Mann–Whitney U test, and the effect size $r$ below each p-value was calculated as $\left(Z/\sqrt{n}\right)$. HC, healthy controls; MLR, monocyte-to-lymphocyte ratio; MS, multiple sclerosis; NLR, neutrophil-to-lymphocyte ratio; PLR, platelet-to-lymphocyte ratio.

The total and differential WBC counts were subsequently compared between the MS and HC groups. The results are listed in the lower half of Table 1, and the boxplots for each of the measured blood cell counts and their ratios are depicted in Fig. 1. The WBC count was significantly higher in the MS group than in the HC group (Mann–Whitney U test, *p* < 0.0001). The RBC count (*p* = 0.0260) and platelet count (*p* = 0.0155) were not significantly different between the two groups. The differential counts of the five WBC subtypes were evaluated and compared between the groups to further investigate the elevated WBC count in the MS group. The differential counts of monocytes (*p* < 0.0001), basophils (*p* = 0.0027), and neutrophils (*p* < 0.0001) were significantly higher in the MS group than in the HC group, whereas the differential counts of lymphocytes (*p* = 0.5025) and eosinophils (*p* = 0.3523) did not differ significantly between the two groups. Further derivatives of NLR, PLR, and MLR were achieved using each of these components for the WBC count. The NLR (*p* = 0.0029) and MLR (*p* = 0.0003) were significantly higher in the MS group than in the HC group, whereas the PLR was the same in both groups. For reference, the blood neutrophil counts of the two patients with primary progressive MS were 3550 and 3050 cells/μL, and the blood monocyte counts were 290 and 340 cells/μL, respectively.

The ANCOVA, using the number of daily cigarette consumption and BMI, was further performed for each WBC differential to adjust for the possible confounding effects of the current smoking status and obesity on the measured WBC differentials. As a result, the monocyte (*p* < 0.0001), basophil (*p* = 0.0009), and neutrophil (*p* < 0.0001) counts remained significantly higher in the MS group than in the HC group, even after adjusting for these potential confounding factors. Meanwhile, the eosinophil (*p* = 0.0297) and lymphocyte (*p* = 0.4130) counts still showed no difference between the groups, even after adjusting for these factors.

### Correlations between differential counts of WBC subtypes

**Figure 2:**
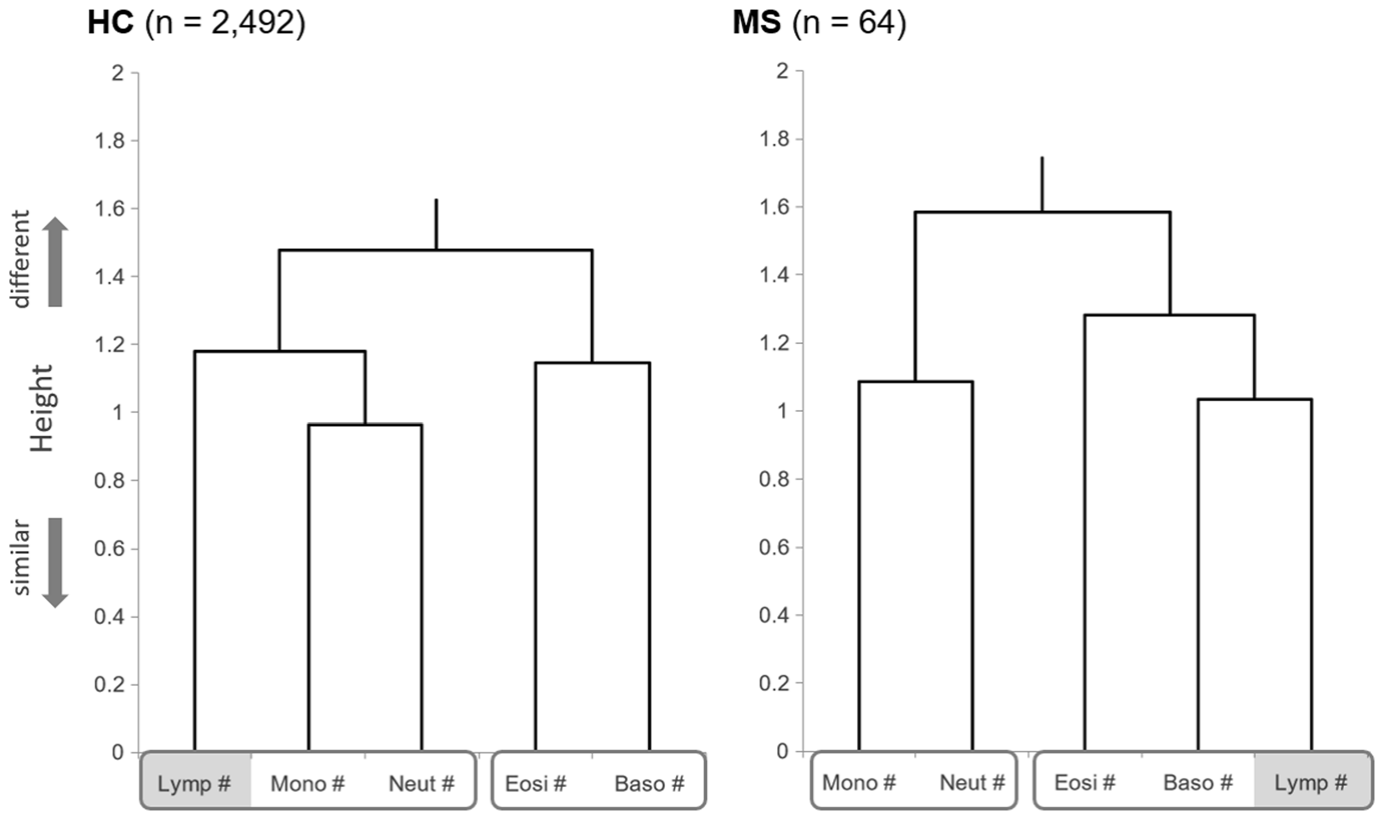
Dendrograms generated from Hierarchical Clustering of White Blood Cell Subtypes in the HC and MS Groups. The dendrogram generated for the HC group was with the distance of the lymphocyte count closer to monocyte and neutrophil counts than to eosinophil or basophil counts. Meanwhile, the dendrogram showed a different structure in the MS group, with the distance of the lymphocyte count closer to eosinophil and basophil counts. HC, healthy controls; MS, multiple sclerosis.

Next, the correlation coefficients between each pair of differential counts of the five WBC subtypes were evaluated (Table 2). The correlation between neutrophils and lymphocytes was confirmed in the HC group (R =  + 0.329). Meanwhile, there was no correlation in the MS group (R = − 0.096). The difference in the correlation coefficient between the HC and MS groups was statistically significant (*p* = 0.0007). The correlation between monocytes and lymphocytes was present in the HC group (R =  + 0.395), but was absent in the MS group (R =  + 0.099). Conversely, the correlation between basophils and lymphocytes was absent or very weak in the HC group (R =  + 0.195), whereas it was statistically significant in the MS group (R =  + 0.467). Correlation coefficients for other pairs of WBC subtypes were not significantly different between the HC and MS groups. Dendrograms for the five differential counts in the HC and MS groups were constructed using hierarchical clustering to visually confirm the different features of the correlation network between the WBC subtypes in the MS group (Fig. 2). The generated dendrograms showed different structures and abnormal lymphocyte behavior in the MS group. The structures of the dendrogram for the other four white blood cell subtypes in the MS group were similar to those in the HC group.

**Table 2:** Correlation matrix between white blood cell subtypes in patients with MS and healthy controls.

	Lymphocyte	Monocyte	Eosinophil	Basophil	Neutrophil
**HC (n = 2492)**					
Lymphocyte	–	R = + 0.395*	R = + 0.257*	R = + 0.195*	R = + 0.329*
Monocyte	(*p* < 0.0001)	–	R = + 0.241*	R = + 0.158*	R = + 0.535*
Eosinophil	(*p* < 0.0001)	(*p* < 0.0001)	–	R = + 0.344*	R = + 0.153*
Basophil	(*p* < 0.0001)	(*p* < 0.0001)	(*p* < 0.0001)	–	R = + 0.126*
Neutrophil	(*p* < 0.0001)	(*p* < 0.0001)	(*p* < 0.0001)	(*p* < 0.0001)	–
**Patients with MS (n = 64)**					
Lymphocyte	–	R = + 0.099	R = + 0.193	R = + 0.467*	R = –0.096
Monocyte	(*p* = 0.4380)	–	R = + 0.117	R = + 0.241	R = + 0.412*
Eosinophil	(*p* = 0.1260)	(*p* = 0.3585)	–	R = + 0.311	R = + 0.162
Basophil	(*p* = 0.0001)	(*p* = 0.0553)	(*p* = 0.0125)	–	R = + 0.105
Neutrophil	(*p* = 0.4501)	(*p* = 0.0007)	(*p* = 0.2002)	(*p* = 0.4098)	–
**Difference in correlation coefficients between HC and MS groups**					
Lymphocyte	–	*p* = 0.0139	*p* = 0.6039	*p* = 0.0173	*p* = 0.0007*
Monocyte	(z = 2.461)	–	*p* = 0.3214	*p* = 0.5055	*p* = 0.2191
Eosinophil	(z = 0.519)	(z = 0.992)	–	*p* = 0.7721	*p* = 0.9426
Basophil	(z = –2.380)	(z = –0.666)	(z = 0.290)	–	*p* = 0.8695
Neutrophil	(z = 3.382)	(z = 1.229)	(z = –0.072)	(z = 0.164)	–

Pearson’s correlation coefficients (R) for each pair of white blood cell subtypes in HC and MS patients are shown. At the bottom of the table, statistical differences for each calculated R between the HC and MS groups are listed, together with the calculated Fisher’s $z$ for each pair.

HC, healthy control; MS, multiple sclerosis.

*p < 0.005.

### Scatterplots of lymphocyte and neutrophil counts in the HC and MS groups

**Figure 3:**
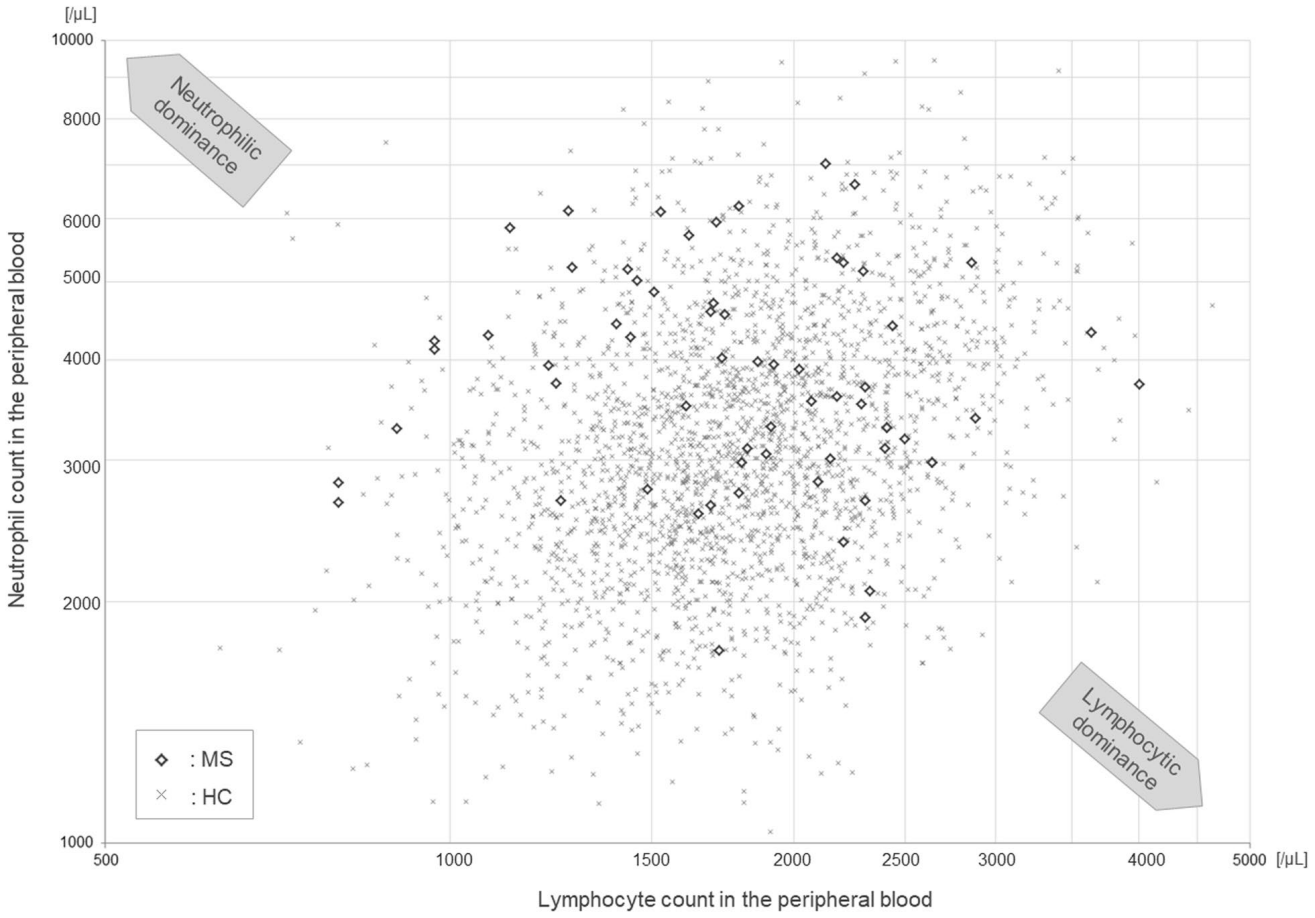
Scatterplot of Lymphocyte and Neutrophil Counts in the Blood of the HC and MS Groups. Patients with MS showed a significantly higher distribution of neutrophil-to-lymphocyte ratio, together with no correlation between neutrophil and lymphocyte counts, compared to those in HC. Note that both the vertical and horizontal axes are log-transformed. HC, healthy controls; MS, multiple sclerosis.

The correlation between the differential counts of the neutrophil and lymphocyte pairs (R =  + 0.329 vs. − 0.096, *p* = 0.0007) was significantly different between the HC and MS groups. Thus, scatterplots depicting the differential counts of neutrophils and lymphocytes were further constructed for the HC and MS groups (Fig. 3). On the log-transformed scatterplot, the distribution in MS patients was largely dispersed compared to that in HC, mainly in the upper-left area of the graph (i.e., suggesting neutrophilic dominance). Fifteen of the 64 patients with MS (23.4%) showed NLR values higher than the values for 95% of the individuals in the HC group. The correlation between neutrophil and lymphocyte counts was apparent in the HC group, whereas it was not apparent in the MS group.

### Clinical significance of differential WBC counts in MS

Correlations between the evaluated laboratory data and the variables with respect to the disease activity or severity were further evaluated based on the significantly elevated WBC, monocyte, basophil, and neutrophil counts in MS at the time of diagnosis before starting the treatment (Table 3). None of the evaluated laboratory markers, including the differential WBC counts, significantly correlated with the disease activity (number of T2 hyperintense brain lesions) or severity (EDSS score after 1 year), except for a negative correlation between the eosinophil count and the number of T2 hyperintense brain lesions. Even when the impact of each differential WBC on disease activity or severity was evaluated based on its percentage [%] (i.e., not the absolute count [/μL]), none of the five cell types showed a significant impact on the disease activity or severity. For reference, the monocyte count showed a weak positive correlation with the value of QAlb (Pearson’s R =  + 0.397, *p* = 0.0039), whereas the other WBC differentials did not.

**Table 3:** Correlations between the evaluated variables and disease activity or severity in MS.

	Numbers of T2 hyperintense brain lesions	The EDSS score after 1 year from the hemogram
Age	rho = + 0.301 (*p* = 0.0166)	rho = + 0.397 (*p* = 0.0012)
Onset phenotype with optic neuritis	rho = 0.052 (*p* = 0.6832)	rho = − 0.298 (*p* = 0.0169)
Onset phenotype with acute myelitis	rho = − 0.115 (*p* = 0.3704)	rho = + 0.441 (*p* = 0.0003)
Total WBC count	rho = − 0.018 (*p* = 0.8886)	rho = − 0.006 (*p* = 0.9622)
RBC count	rho = − 0.225 (*p* = 0.0762)	rho = − 0.094 (*p* = 0.4622)
Platelet count	rho = + 0.147 (*p* = 0.2496)	rho = + 0.141 (*p* = 0.2670)
Monocyte count	rho = − 0.090 (*p* = 0.4850)	rho = − 0.098 (*p* = 0.4412)
Lymphocyte count	rho = − 0.096 (*p* = 0.4550)	rho = − 0.053 (*p* = 0.6751)
Eosinophil count	rho = − 0.447 (*p* = 0.0002)	rho = − 0.120 (*p* = 0.3470)
Basophil count	rho = + 0.074 (*p* = 0.5635)	rho = + 0.039 (*p* = 0.7570)
Neutrophil count	rho = + 0.077 (*p* = 0.5475)	rho = + 0.027 (*p* = 0.8339)
PLR	rho = + 0.143 (*p* = 0.2648)	rho = + 0.160 (*p* = 0.2081)
MLR	rho = − 0.027 (*p* = 0.8335)	rho = − 0.005 (*p* = 0.9689)
NLR	rho = + 0.095 (*p* = 0.4598)	rho = + 0.042 (*p* = 0.7393)
CSF cell count	rho = − 0.043 (*p* = 0.7523)	rho = − 0.094 (*p* = 0.4924)
OCB positivity	rho = + 0.132 (*p* = 0.3335)	rho = + 0.120 (*p* = 0.3760)
IgG-index	rho = + 0.197 (*p* = 0.1585)	rho = + 0.249 (*p* = 0.0721)
QAlb	rho = − 0.009 (*p* = 0.9509)	rho = + 0.219 (*p* = 0.1234)
QIgG	rho = + 0.087 (*p* = 0.5425)	rho = + 0.318 (*p* = 0.0228)

Spearman’s correlation coefficients (rho) and the *p* values of the test of no correlation between the clinical manifestations and laboratory test data in MS are shown.

CSF, cerebrospinal fluid; EDSS, Kurtzke Expanded Disability Status Scale; IgG, immunoglobulin G; MLR, monocyte-to-lymphocyte ratio; NLR, neutrophil-to-lymphocyte ratio; OCB, oligoclonal bands; PLR, platelet-to-lymphocyte ratio; QAlb, quotient of albumin; QIgG, quotient of immunoglobulin G; RBC, red blood cell; WBC, white blood cell.

## Discussion

The profiles of blood cell counts in the peripheral blood of patients with MS before starting acute or chronic treatment were comprehensively evaluated and compared with the profiles of HCs in this study. The results revealed that the total WBC count and differential monocyte, basophil, and neutrophil counts, all of which are related to the innate immune system, were significantly elevated in the MS group compared to the HC group. Meanwhile, the lymphocyte count was not elevated in the MS group. The correlation between neutrophil and lymphocyte counts was apparent in the HCs, but was not apparent in the MS group. Based on the results that none of the patients tested positive for the serum antibodies against major viral infections (e.g., HBV, HCV, HIV, HSV, VZV), all the clinical attacks at the time of the hemogram in the 64 patients with MS could be considered to have occurred in the absence of infection. Moreover, the resultant findings were irrespective of the current amount of cigarette consumption or the BMI. These results suggest that the balance between the innate and adaptive (i.e., acquired) immune system may have changed systemically in MS from the early stages, which could have resulted in the observed elevations in NLR and MLR. An elevated NLR in patients with MS compared to healthy individuals has been similarly reported in several previous studies^6,7^. These previous studies even demonstrated that the level of NLR may affect the subsequent disability progression. These facts indicate that an upregulated innate immunity may play a possible role in some steps of the disease development and progression in MS.

Meanwhile, it has been known that an upregulated innate immune system can be observed non-specifically in several conditions. In general, neutrophils are the key cell type of the innate immune system, as well as the first cellular defense against external pathogens, including infections. Neutrophil trafficking to the site is an important step of inflammation, constituting a hallmark of inflammation, and presents as an increase in neutrophil and a decrease in lymphocyte counts. The inflammatory response is considered to result in the production of neutrophils and accelerated apoptosis of lymphocytes^14^. Consequently, the finding of the elevated total WBC count with elevated NLR would not certainly be distinct to MS, and the achieved results could have resulted from a non-specific activation of the innate immune system with the mild suppression of adaptive immunity. In neurological conditions other than MS, an elevated NLR and/or its potential for outcome prediction has been suggested in stroke, and some neurodegenerative disorders, such as Alzheimer’s and Parkinson’s diseases^15–17^. A shift from adaptive to upregulated innate immune systems may play some role in the progression or acceleration of the neurodegenerative processes in the damaged site in these diseases. Furthermore, this study failed to show significant positive correlations between the elevated differential WBC counts and the markers of disease activity or severity in MS. The exact role of elevated blood neutrophil and monocyte counts in the development and progression of MS remains largely unknown. A conceivable theory may be that psychological stress due to the neurological disturbances among patients with MS could have changed the balance of innate and adaptive immunities, thus resulting in the elevation of NLR and MLR. Psychological stress is known to increase the total WBC, neutrophil, and monocyte counts in the general population^18–20^. Although this is only a speculation as the stress level was not evaluated in the enrolled patients, such psychological distress with possible post-stress inflammation in the blood among the patients with MS could have produced the apparent difference in the total WBC, neutrophil, and monocyte counts between the patients with MS and HCs in this study.

Although the finding of the elevated blood neutrophils and monocytes is not specific to MS and may not play a pivotal role in the development of MS lesions, the role of neutrophils in the development of MS has been long discussed in previous studies. The possible association between neutrophils and the development of demyelinating lesions in MS has been previously reported. Previous studies using an animal model of MS, known as experimental autoimmune encephalomyelitis (EAE), demonstrated that neutrophils play a role in the development of demyelinating lesions by promoting parenchymal infiltration of leukocytes in the CNS^21^. Another study that evaluated the peripheral blood of patients with MS showed that the neutrophils in patients with MS are in a primed state, rather than a resting state, possibly resulting from the chronic proinflammatory environment and resulting in enhanced innate immunity responses during infections^22^. In addition to neutrophils, monocytes have been reported to play a role in the pathogenesis of MS. A previous study on EAE showed that circulatory monocytes prefer proinflammatory M1 activation rather than the immunomodulatory M2 activation and this plays a key role in the development of demyelinating CNS lesions^23^. Several other previous reports on peripheral blood samples from patients with MS demonstrated that elevated monocytes in MS are possibly associated with disease severity or subsequent neurological prognosis^7,24^. Meanwhile, neutrophils are not the major cell type of parenchymal infiltrations in MS lesions. Together with the results of the present study, these facts suggest that an activated innate immune system based on a chronic proinflammatory environment underlies the pathogenesis of MS and promotes cellular parenchymal infiltrations through the blood–brain barrier, which result in disease relapses. Furthermore, the results of this study and previous studies imply the possible role of the activated or primed innate immune system in MS may play roles in MS disease progression and the indolent progression of neurological disturbances between relapses. The current relapse-prevention treatments for MS mainly focus on suppressing the functions of the adaptive immune system, but suppressing the innate immune system in MS may be a possible therapeutic option.

This study has some limitations. First, the sample size in the MS group was relatively small. However, if we increase the number of facilities to achieve a larger sample size, the risks of bias based on errors between facilities may also increase. As such errors between facilities would surely cause significant bias in the measured WBC differential counts and the calculated correlation coefficients between the five WBC subtypes (especially for white blood cells with low counts such as eosinophils and basophils), the present study was performed using data from a single facility. Another limitation was that the mechanism and physiological significance of the observed correlation between lymphocyte and basophil counts in the MS group is unknown. Currently, the exact role of basophils in the pathogenesis of autoimmune-related diseases is not fully elucidated, although it is believed to be associated with innate immunity and plays a role in chronic allergic inflammations^25^. To establish the significance of monocytes, basophils, and neutrophils in the pathogenesis of MS, further research is needed to clarify the mechanisms underlying the altered profiles of these blood cells in patients with MS.

In conclusion, patients with MS are likely to show elevated counts of neutrophils, monocytes, and basophils in the peripheral blood at the early disease stages before the initiation of acute or chronic treatment. A positive correlation between the neutrophil and lymphocyte counts, which was evident in the healthy population, was weakened in patients with MS. A systemic shift from adaptive to upregulated innate immune processes may play possible role in the development or progression of MS and necessitates the need for further research with respect to the role of neutrophils and monocytes in the pathophysiology of MS.

## Data Availability

Data regarding the blood test data for MS patients are available from the authors on reasonable requests from qualified clinicians or researchers.

## Abbreviations

HC: Healthy controls
MLR: Monocyte-to-lymphocyte ratio
MS: Multiple sclerosis
NLR: Neutrophil-to-lymphocyte ratio
PLR: Platelet-to-lymphocyte ratio
WBC: White blood cell
